# Simultaneous acoustic stimulation of human primary and secondary somatosensory cortices using transcranial focused ultrasound

**DOI:** 10.1186/s12868-016-0303-6

**Published:** 2016-10-26

**Authors:** Wonhye Lee, Yong An Chung, Yujin Jung, In-Uk Song, Seung-Schik Yoo

**Affiliations:** 1 Incheon St. Mary’s Hospital, The Catholic University of Korea, Incheon, Republic of Korea; 2 Department of Radiology, Brigham and Women’s Hospital, Harvard Medical School, Boston, MA USA

**Keywords:** Dual transcranial focused ultrasound, Image-guidance, Non-invasive brain stimulation, Human primary and secondary somatosensory cortices, Tactile sensations

## Abstract

**Background:**

Transcranial focused ultrasound (FUS) is gaining momentum as a novel non-invasive brain stimulation method, with promising potential for superior spatial resolution and depth penetration compared to transcranial magnetic stimulation or transcranial direct current stimulation. We examined the presence of tactile sensations elicited by FUS stimulation of two separate brain regions in humans—the primary (SI) and secondary (SII) somatosensory areas of the hand, as guided by individual-specific functional magnetic resonance imaging data.

**Results:**

Under image-guidance, acoustic stimulations were delivered to the SI and SII areas either separately or simultaneously. The SII areas were divided into sub-regions that are activated by four types of external tactile sensations to the palmar side of the right hand—vibrotactile, pressure, warmth, and coolness. Across the stimulation conditions (SI only, SII only, SI and SII simultaneously), participants reported various types of tactile sensations that arose from the hand contralateral to the stimulation, such as the palm/back of the hand or as single/neighboring fingers. The type of tactile sensations did not match the sensations that are associated with specific sub-regions in the SII. The neuro-stimulatory effects of FUS were transient and reversible, and the procedure did not cause any adverse changes or discomforts in the subject’s mental/physical status.

**Conclusions:**

The use of multiple FUS transducers allowed for simultaneous stimulation of the SI/SII in the same hemisphere and elicited various tactile sensations in the absence of any external sensory stimuli. Stimulation of the SII area alone could also induce perception of tactile sensations. The ability to stimulate multiple brain areas in a spatially restricted fashion can be used to study causal relationships between regional brain activities and their cognitive/behavioral outcomes.

**Electronic supplementary material:**

The online version of this article (doi:10.1186/s12868-016-0303-6) contains supplementary material, which is available to authorized users.

## Background

Brain stimulation techniques serve as important tools for neurotherapeutics and allow for functional investigation of the brain [1, 2]. Methods such as deep brain stimulation (DBS) or epidural cortical stimulation (EpCS) have been utilized in clinical settings for the treatment of neurological or neuropsychiatric diseases [2], but these techniques involve invasive surgical procedures. Non-invasive techniques such as transcranial magnetic stimulation (TMS) or transcranial direct current stimulation (tDCS) are available to modulate neural functions without surgery [1, 3], but the stimulatory area is relatively large (on the order of centimeters) and its depth is limited proximal to the cortical surface [2, 4]. Optogenetic approaches offer cell-level modification of neuronal excitability [5, 6]; however, the required introduction of genetic alterations to introduce sensitivity to light prohibits immediate applications in humans. Therefore, the development of non-invasive and spatially-selective means of brain stimulation was sought after.

Focused ultrasound (FUS) has recently shown its utility in non-invasive brain stimulation [7], with greater spatial selectivity and depth penetration compared to other non-invasive techniques such as TMS or tDCS [8–10]. The acoustic neuromodulatory effects can be tailored as either excitatory or suppressive, depending on the sonication parameters [11, 12]. Accumulating ex vivo [13, 14] and in vivo [12, 15–18] evidence shows that acoustic pressure waves delivered to localized brain structures modulate their excitability using low-level acoustic intensity (i.e., compatible with potential human application [19, 20]). Recently, transcranial FUS has also been shown to have neuromodulatory effects on large animal models, such as the elicitation of motor and visual electrophysiological responses in sheep [21] and the modulation of saccadic movement in non-human primates [22]. In humans, transcranially delivered FUS to the primary somatosensory cortex (SI) has been shown to modulate the performance of tactile discrimination tasks as well as the amplitude of somatosensory evoked potentials (SEP) [19]. More recently, we have demonstrated that FUS sonication of the SI, without giving external sensory stimulation, evoked both sonication-specific electroencephalographic (EEG) responses and various tactile sensations from the hand area [20].

In addition to the SI (a primary site of processing external sensory afferent signals), the SII (located in the parietal operculum on the ceiling of the lateral sulcus) is an important neural substrate for processing/cognition of various tactile sensations, including pain or even visceral sensations [23, 24]. To our knowledge, studies on the stimulation of the SII areas in humans are rare. Spatial specificity of FUS confers the ability to simultaneously stimulate multiple brain regions that are close to each other, whereas the concurrent operation of multiple TMS coils in close proximity is not desirable due to the mutual interactions/interferences of the magnetic fields [25]. Only limited TMS studies have been reported to stimulate brain areas, one from each hemisphere [26], or to stimulate adjacent brain regions with temporal gaps in between [25]. Therefore, we were motivated to deliver neurostimulatory FUS to the SII, and to examine the outcomes in terms of subjective sensations felt by the individuals. The existence of spatially-distinct sub-regions within the SII for processing different types of tactile sensations [23, 27] prompted us to further explore the possibility that FUS stimulation of sensation-specific SII areas (i.e., vibrotactile, pressure, warmth, and coolness) may also induce corresponding types of tactile sensations. The FUS was also administered to both SI and SII simultaneously, and its effects were assessed.

## Methods

### Participants and study overview

This research was performed under the approval of the Institutional Review Board (IRB) of Incheon St. Mary’s Hospital, the Catholic University of Korea, in accordance with the ethical guidelines set forth by the IRB. Ten healthy volunteers (two females, ages 23–34, average of 27.8 ± 4.1 years, labeled ‘h1’ through ‘h10’ herein) with no clinical history of peripheral/central neurological diseases participated. All participants submitted written consent prior to enrollment in the study.

Prior to the FUS procedures, functional MRI (fMRI) was performed (on a separate day) to map the individual-specific SI and SII areas in the left hemisphere that are functionally eloquent for four different non-painful sensory stimuli—(1) vibrotactile, (2) pressure, (3) warmth, and (4) coolness [27]. Anatomical MRI and computed tomography (CT) scans of the head were also acquired on the same day. The acquired neuroimage data were used for neuroradiological assessments, such as, but not limited to, existence of clinically significant intracranial calcifications (mainly detected by the CT), which may disturb the acoustic propagation within the cranial cavity (none were found). Along with the MRI/CT procedures, clinical neurological examination and the mini-mental state examination (MMSE) [28] were provided to each subject by licensed physicians.

The FUS procedures, conducted on a separate day (gap between the MRI/CT and FUS procedures: 98.7 ± 6.0 days; mean ± SD, *n* = 10), were divided into multiple sessions—(1) stimulation of the SI alone (i.e., SI_FUS_), (2) stimulation of four sub-regions in the SII (i.e., SII_FUS_; in which the coordinates corresponding to the four types of tactile stimuli were identified), (3) stimulation of both SI and SII (i.e., SI/SII_FUS_; four different SII regions were stimulated), and (4) sham condition (i.e., Sham_FUS_, using the same FUS setup as SI/SII_FUS_, but without delivery of any sonication). The sequence of these stimulation conditions was randomized and balanced across all subjects. Additional neurological examination and MMSE were administered on the day of the sonication experiments both before and after FUS administration to examine the presence of any neurological changes.

### Multi-modal imaging data and sonication planning

Both CT and anatomical MRI of the participants’ head was used for planning and image-guidance of FUS sonication [20]. Adhesive fiducial markers (PinPoint; Beekly Corp., Bristol, CT; visible in both MRI and CT) were attached on four locations spatially distributed over the head. Since these adhesive fiducial markers were also used for image-guidance of the sonication (that was conducted in a separate day), their reproducible positioning was crucial. To do so, we carefully identified the participants permanent anatomical features, such as skin imperfections (such as wrinkle lines and/or spots) or skin vein structures (such as bifurcation) to place the markers (on them). These sites were photographed to be used for later positioning. The spatial coordinates of these markers in the acquired CT/MRI data were utilized as a basis for the spatial co-registration between neuroimage space and the physical location of the subject’s head.

A clinical CT scanner (Aquilion ONE, Toshiba, Japan) was used to acquire the CT data of the head [axial orientation, slice thickness = 0.5 mm, field-of-view (FOV) = 24 × 24 cm^2^, image matrix = 512 × 512, voxel size = 0.47 × 0.47 × 0.50 mm^3^]. The head CT data were used to plan for the orientation of the transcranial FUS, whereby we aligned the sonication pathway as perpendicular as possible to the skull at the entry, while avoiding thick skull segments or in-bone air-pockets (both significantly distort the acoustic beam propagation by attenuation and diffraction/reflection). To obtain the head MRI data, a 3-Tesla clinical MR scanner (MAGNETOM Skyra, Siemens) was utilized with a 4-channel head coil. T1-weighted images of the anatomical MRI [3D GRAPPA sequence, acceleration factor = 2, repetition time (TR) = 1900 ms, echo time (TE) = 2.46 ms, flip angle = 9°, FOV = 24 × 24 cm^2^, image matrix = 256 × 256, slice thickness = 0.94 mm, voxel size = 0.94 × 0.94 × 0.94 mm^3^, sagittal orientation, 192 slices] were acquired from the head, covering the entire telencephalic areas. Then, blood oxygenation level dependent (BOLD)-fMRI was conducted for each subject to map the individual-specific SI and SII areas, functionally eloquent for four different tactile stimulations of the right hand—(1) vibrotactile, (2) pressure, (3) warmth, and (4) coolness. The detailed stimulation paradigm for the fMRI and the image processing schemes are reported elsewhere [27].

The functional and anatomical MRI data, as well as the cranial information from the CT scan, were spatially co-registered (using the Normalized Mutual Information technique [29]), and these multi-modal imaging data were utilized for the planning and on-site individual-specific neuroimage-guidance for transcranial FUS sonication [20]. Individual-specific coordinates of the SI and SII in the left hemisphere were identified based on our previous study on the same participants [27]. Within the SI, local maxima of the activations corresponding to different tactile stimuli were closely clustered and overlapped each other; therefore, a single sonication target was assigned representing the SI area. On the other hand, the locations of activation in the SII associated with different tactile stimuli showed a degree of spatial distributions (having a radius of 5.3 ± 2.6 mm; as identified from the local maximum in the activation probability) while a degree of individual variability existed (i.e., ranged from 2.1 to 10.3 mm; a group-level spatial distribution of the SII sub-regions was described elsewhere [27]). Thus, the SII areas were divided into four different spatial locations to be targeted by FUS.

### The sonication setup

**Fig. 1:**
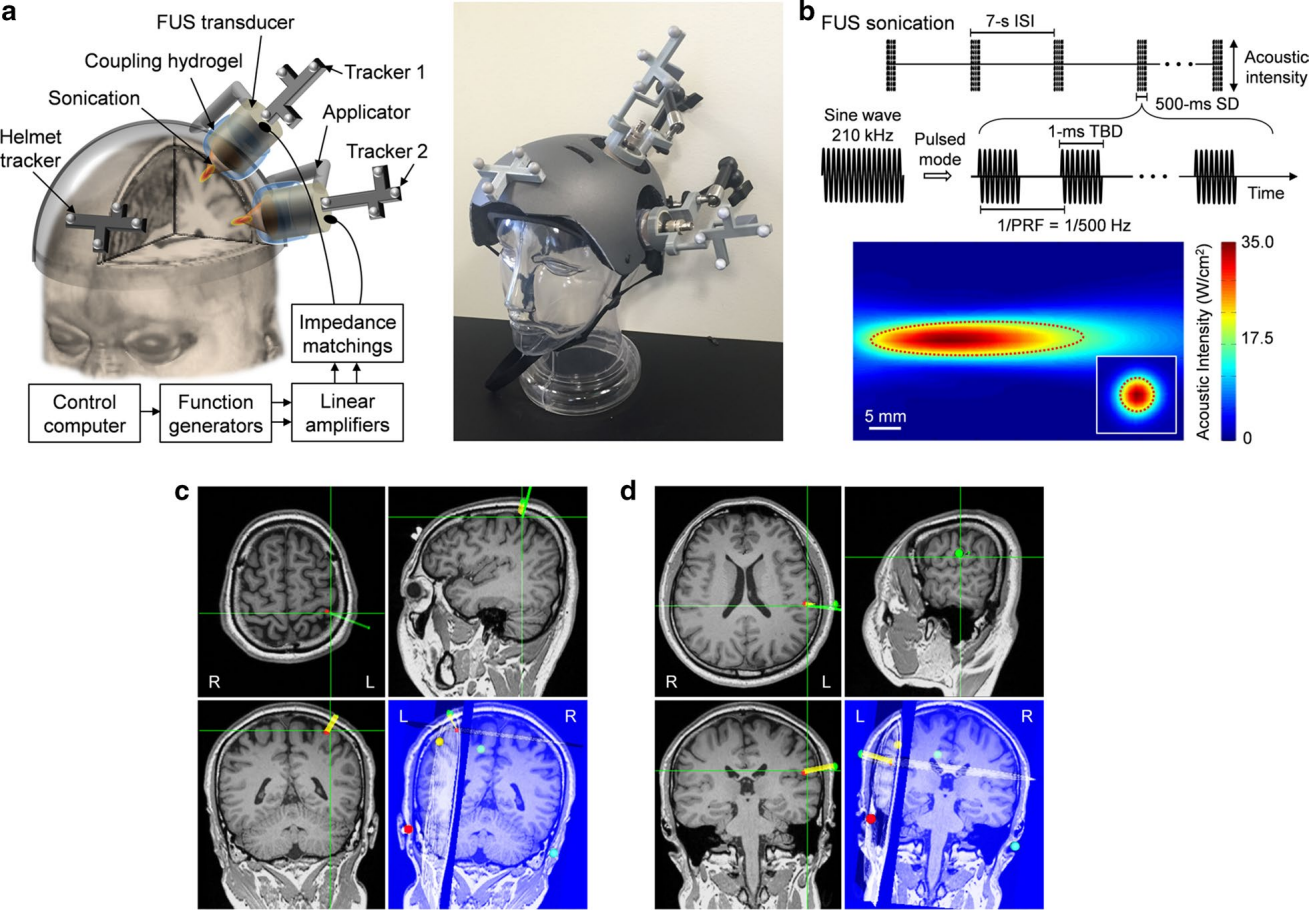
Experimental schematics of the dual FUS application with the sonication parameters. **a** *Left panel* a rendering of the FUS setup, and *right panel* its actual implementation on a mannequin head model. The two FUS foci were placed at the targeted SI and SII by image-guidance using optical trackers (‘tracker 1’ and ‘tracker 2’) in reference to the subject head (tracked via ‘helmet tracker’). Each tracker had four infrared-reflective markers for real-time motion detection. FUS transducers were actuated by the sinusoidal electrical signals with impedance matching circuits. Compressible hydrogel was used to couple the FUS transducer to the scalp. **b** *Upper panel* illustration of the acoustic parameters. *SD* sonication duration = 500 ms, *ISI* inter-stimulation-interval = 7 s, *TBD* tone-burst-duration = 1 ms, *PRF* pulse-repetition-frequency = 500 Hz; Incident spatial-peak pulse-average intensity = 35.0 W/cm^2^ I_sppa_. *Lower panel* acoustic intensity mapping of the 210 kHz FUS transducer (longitudinal measurement was taken 10 mm from the exit plane of the transducer). The *red dotted lines* indicate the FWHM of the intensity profile. **c**, **d** Exemplar views of the individual-specific neuroimage-guidance for targeting of ipsilateral SI or SII, respectively. The green crosshairs shown in the projection views (i.e., axial, sagittal, and coronal slices) indicates the sonication target, and the *thick green line* and *yellow line* represent the orientation of the sonication path and planned path, respectively, connecting the target (*red dot*) and entry (*green dot*) points. In the *lower right panel*, the four *colored dots* (without the *yellow bar*) show the locations of anatomical markers used for the neuroimage-registration with the subject. *R* and *L* denote right and left, respectively

In order to independently deliver acoustic energy to the SI and SII in the left hemisphere, we used two sets of single-element FUS transducers (operating at 210 kHz frequency, The Ultran Group Ltd, State College, PA) (Fig. 1a), which were segmented-spheres in shape, each having an outer diameter (OD) of 30 mm and a focal distance of 25 mm. Each transducer was affixed to an articulated applicator (Zamerican, Zacuto, Chicago, IL) that was mounted on a helmet (named ‘FUS helmet’, Fig. 1a, modified from Giro Section Helmet, Santa Cruz, CA) having two open spaces (8 cm in diameter) to allow access to the SI and SII in the left hemisphere. The position and orientation of the transducers could be adjusted and locked using the applicators. The gap between the scalp and the transducer surface was filled with a polyvinyl alcohol (PVA) hydrogel for acoustic coupling. The compressible PVA hydrogel (having a thickness of ~10 mm) which was fitted around the transducer allowed for adjustment of acoustic focal depth in the range of 5–20 mm (detailed implementation was described elsewhere [30]). The subject’s hair was parted in the middle of each sonication entry point, and a generic ultrasound hydrogel (Aquasonics, Parker Laboratories, Fairfield, NJ) was applied onto the exposed scalp.

For image-guided alignment of the FUS focus to the intended target, the relative location and orientation of the transducers with respect to the helmet (i.e., subject’s head) were tracked in real-time, whereby the coordinates of the focus can be visualized on the individual-specific neuroanatomy (as well as the planned sonication target) via a custom-built image-guidance system as previously described [20, 31]. An optical tracker was attached to the helmet and each of the two FUS transducers for motion tracking. Each FUS transducer was actuated by a computer-controlled driving circuit (Fig. 1a). Two sets of the driving circuits were used to actuate each of two FUS transducers. Upon receiving a trigger signal from the control computer, the input signal (Fig. 1b) was generated by a pair of function generators (33220A; Agilent technologies, Inc., Santa Clara, CA) and amplified by a Class-A linear power amplifier (Electronics and Innovations, Rochester, NY). An impedance-matching circuit was used to increase the power efficiency.

### Operating parameters and characterization of the FUS acoustic field

Based on our previous experiences [20, 21], 210 kHz ultrasound was used to achieve an effective acoustic transmission through the thick skull. We adapted similar sonication parameters that were utilized in the successful stimulation of the SI in humans [20] and in animals [12, 16, 21] (Fig. 1b), having a sonication duration (SD) of 500 ms, with a tone-burst-duration (TBD) of 1 ms repeated at a frequency of 500 Hz (i.e., pulse repetition frequency; PRF), yielding a 50% duty cycle. The spatial profile of the acoustic intensity field generated by the FUS transducer was characterized (Fig. 1b) using methods described elsewhere [12]. The diameter of the FUS focus was measured on the acoustic intensity maps based on pressure scanning using a hydrophone (HNR500; Onda, Sunnyvale, CA) over the transversal plane (31 × 31 mm^2^ square area, 1 mm step) perpendicular to the sonication path at the acoustic focal distance using time-of-flight information. The length of the focus was measured along the longitudinal plane along the beam path (31 × 51 mm^2^ area, 1 mm step, measured 10 mm away from the exit plane of the transducer). The acoustic focus had a diameter of 6 mm and a length of 38 mm, as defined by the full-width at half-maximum (FWHM) of the acoustic intensity map (Fig. 1b). The incident acoustic intensity at the FUS focus, in the absence of skull, was 35.0 W/cm^2^ spatial-peak pulse-average acoustic intensity (I_sppa_), resulting in a spatial peak temporal-average acoustic intensity (I_spta_) of 17.5 W/cm^2^.

### Image-guided FUS to the primary and secondary somatosensory cortices

On the day of the sonication experiment, the subject was seated in a recliner chair. Prior to the spatial registration of the subject’s physical space to the virtual space of the head MRI/CT neuroimage data, fiducial markers (stickers) were attached to the same locations that were used for the sonication planning (i.e., during the initial MRI/CT session). The registration quality was assessed to minimize target registration error (TRE) [32, 33], which was less than 4 mm (3.7 ± 1.4 mm, *n* = 10, mean ± SD). The FUS helmet was then tightly secured on the subject’s head to maintain the location of the transducer with respect to head motion. A set of optical trackers attached to the helmet (‘helmet tracker’ in Fig. 1a) and transducers (‘tracker 1’ and ‘tracker 2’, in Fig. 1a) provided the orientation and location of the acoustic foci back to the experimenters, following the methods described in our previous work [20]. Under this image-guidance, the experimenters aligned the FUS focus to the intended coordinates of the somatosensory areas (Fig. 1c, d). The orientation of the sonication path was adjusted to make the incident angle as perpendicular as possible to the scalp (at an entry point), as guided by the information established during the sonication planning stage (see “Multi-modal imaging data and sonication planning” section).

The alignment of FUS foci was repeated prior to the beginning of each session (i.e., vibrotactile, pressure, warmth, and coolness), and the sonication was administered 20 times for each session across the conditions (i.e., SI_FUS_, SII_FUS_, SI/SII_FUS_, and Sham_FUS_). The participants were instructed to tap a touch sensor on their left index finger (pulse transducer MLT1010/D; ADInstruments, CO) to report the timing of the tactile sensation during the sonication experiment, and also to verbally report the location and type of the sensations upon the completion of each stimulation condition within the FUS session. Both the subject and the operator were blinded to the nature of the sonication (i.e., the intended elicitation of the tactile sensation including its side). The subject’s tapping response and the timing of the sonication events were measured using the data acquisition system (LabChart 7 and PowerLab 4/35; ADInstruments).

### Post-FUS session follow-up

After the FUS procedure, subjects were asked to remain in the study premises for 2 h, and received the post-FUS neurological examination and MMSE. Subsequently, anatomical MRI data were acquired again for follow-up neuroradiological examination from all participants at three different time periods—same day (*n* = 3), 2 weeks (*n* = 4), and 4 weeks (*n* = 3) after the sonication session. The physicians who conducted the neurological assessments were blinded to the nature of the study. Two months after the sonication sessions, all subjects were interviewed by telephone to check the presence of any changes regarding mental or physical discomforts/health status concerned with the study participation.

## Results

### Response rate of eliciting sensation by the FUS stimulation

FUS stimulation, via sonication of either the SI/SII separately or both the SI and SII simultaneously, elicited tactile sensations from the subjects whereby the response rate, as defined by the number of reported tactile responses out of 20 stimulation events, are summarized in Table 1. Not all of the FUS stimulation events elicited sensations from the subjects. For example, one subject (‘h10’) did not report any sensation during any of the FUS conditions (noted as ‘NR’). Subject ‘h8’ also did not report any sensation during the SI/SII_FUS_ condition. Furthermore, across the different FUS conditions, we observed several sessions that a few subjects did not report any elicited sensation (Table 1, indicated as NR). Across the sonication sessions with the elicitation of tactile sensations, there was a degree of variability in the response rates among the subjects, ranging from 50 to 100% in one subject (‘h6’) to 10–35% in another subject (‘h1’). Under the sham condition, none of the participants reported any elicited sensations. Peripheral sensations from the scalp, often observed during the administration of TMS [34–36], were not present. The onset of elicited sensation, as measured from the response time acquisition (Additional file 1: Fig. S1), occurred with a delay of ~2 s after the onset of sonication event (1.83 ± 1.31 s; mean ± SD, *n* = 784).

**Table 1:** Response rates of elicited sensations during the FUS procedures

ID	SI	SII				SII_Ave_	SI/SII				SI/SII_Ave_
		V	P	W	C		V	P	W	C	
h1	25%	20%	20%	35%	20%	24%	10%	20%	30%	20%	20%
h2	65%	40%	70%	50%	25%	46%	65%	35%	65%	65%	58%
h3	90%	60%	60%	50%	70%	60%	75%	85%	30%	75%	66%
h4	90%	NR	95%	NR	NR	95%	90%	75%	NR	NR	83%
h5	45%	55%	NR	85%	30%	57%	5%	45%	25%	15%	23%
h6	75%	65%	70%	70%	80%	71%	50%	80%	100%	80%	78%
h7	95%	85%	NR	65%	75%	75%	95%	85%	70%	90%	85%
h8	30%	45%	20%	NR	NR	33%	NR	NR	NR	NR	–
h9	95%	NR	NR	70%	70%	70%	50%	80%	75%	90%	74%
h10	NR	NR	NR	NR	NR	–	NR	NR	NR	NR	–
Mean	68%	53%	56%	61%	53%	59%	55%	63%	56%	62%	61%
SD	28%	21%	30%	17%	26%	22%	34%	26%	29%	32%	26%

The response rates were derived as ‘the number of sonication events that elicited tactile sensations with respect to the number of sonication events (i.e., 20 events).’ For the FUS conditions of SII_FUS_ and SI/SII_FUS_, the rates were tabulated for each sensation-specific session (*V* vibrotactile, *P* pressure, *W* warmth, *C* coolness), along with the average response rate across the sessions shown as a separate column (Ave). Mean and SD were derived without including the non-responsive cases (denoted as ‘NR’)

To qualitatively assess the degree of responses from the SII_FUS_ and SI/SII_FUS_ conditions, the response rates were averaged across only the sessions where a response was reported (SII_Ave_ and SI/SII_Ave_ in Table 1). Comparison among the different sonication conditions showed that the response rates were not significantly different with each other (via *t* test; all *p* > 0.05). The response rate from the SI_FUS_ condition was also similar to those observed from the previous study on the FUS stimulation of the SI [20] (via *t* test, *p* > 0.05). It is notable, however, that about half of the subjects (*n* = 4) in the present study reported high response rates, showing 90–100% during SI_FUS_. In the previous study that stimulated the SI in humans, none of the participants showed 90% or higher response rates [20].

### Type/location of sensations elicited from FUS stimulation

The types of tactile sensations reported by the responsive subjects are shown in (Table 2a; Additional file 1: Table S1) across the different sonication conditions (i.e., SI_FUS_, SII_FUS_, and SI/SII_FUS_). Among the types of sensations reported by the subjects, a ‘tingling’ sensation was dominant across the different FUS conditions, while sensations such as ‘feeling of weak electrical current flow’ and ‘numbness’ were also reported. Other types of sensations, i.e., ‘heaviness/pressure’, ‘coolness’, and ‘brushing’, were also reported, although the occurrence was not frequent. These elicited sensations were in good agreement with the results from our previous investigation of acoustic stimulation of the SI [20], yet the ‘vibrotactile’ and ‘warmth’ sensations were newly recognized from the present study. The stimulation of different locations of the SII sub-regions did not elicit the corresponding/matching tactile sensations. However, two individuals (‘h2’ and ‘h5’) reported sensations that partially matched the intended type of sensations, for example, ‘warmth’ conditions (SII_FUS_ or SI/SII_FUS_; Additional file 1: Table S1). Another participant, ‘h5’, also reported matching ‘vibrotactile’ and ‘pressure’ sensations in the SI/SII_FUS_ condition.

**Table 2:** Number of subjects categorized by type and location of tactile sensations across different sonication conditions

Types of sensations	Sonication conditions					
	SI_FUS_		SII_FUS_		SI/SII_FUS_	
(a)						
Tingling	7/9	78%	7/9	78%	6/9	67%
Heaviness/pressure	2/9	22%	2/9	22%	1/9	11%
Numbness	3/9	33%	4/9	44%	4/9	44%
Feeling of weak	4/9	44%	5/9	56%	6/9	67%
electrical current flow						
Warmth	2/9	22%	2/9	22%	1/9	11%
Coolness	0/9	0%	1/9	11%	0/9	0%
Vibrotactile	1/9	11%	0/9	0%	2/9	22%
Brushing	1/9	11%	0/9	0%	0/9	0%

Locations of sensations	Sonication conditions					
	SI_FUS_		SII_FUS_		SI/SII_FUS_	
(b)						
Hand/finger(s)	7/9	78%	8/9	89%	7/9	78%
Wrist	1/9	11%	3/9	33%	2/9	22%
Forearm	3/9	33%	3/9	33%	3/9	33%
Elbow	2/9	22%	2/9	22%	5/9	56%
Arm	1/9	11%	1/9	11%	1/9	11%
Leg	3/9	33%	0/9	0%	0/9	0%

The number of subjects categorized (a) according to the types of reported sensation descriptors and (b) the locations of sensations across three sonication conditions (SI_FUS_, SII_FUS_, and SI/SII_FUS_) out of 9 responsive subjects (‘h1’–‘h9’). Detailed information of the elicited sensations from each subject can be found in Additional file 1: Tables S1–S3

**Fig. 2:**
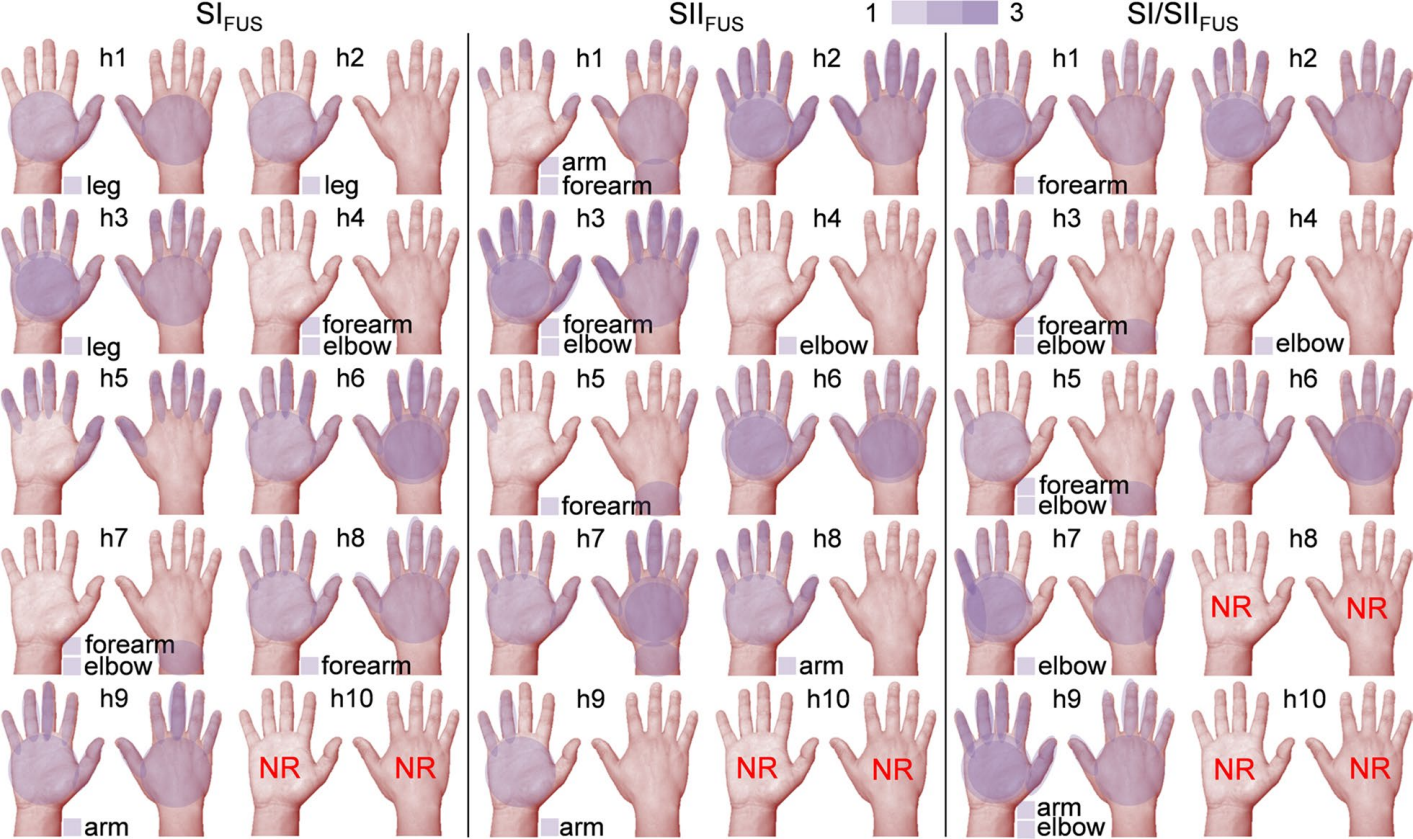
Graphical illustration of the location of tactile sensations. The distinctive locations of the elicited sensations were depicted by semi-transparent *purple color* overlaid on the palmar and dorsal views of the right hand for each subject (‘h1’ through ‘h10’). The additional locations (i.e., wrist, forearm, elbow, arm, and leg) of the elicited sensations were also shown under the hand illustrations. The *left column* shows the locations of the responses during the SI_FUS_ condition. The results from the sensation-specific sessions (i.e., ‘vibrotactile’, ‘pressure’, ‘warmth’, and ‘coolness’) were merged on each column of SII_FUS_ (*middle column*) and SI/SII_FUS_ (*right column*), respectively. The number of occurrences for a set of distinctive locations of a sensation is represented by a *color* scale (*1*–*3*). *NR* non-responsive cases (‘h8’ under the SI/SII_FUS_ condition and ‘h10’ during all FUS procedures)

Across all sonication conditions, the responsive subjects reported the elicited sensations mostly from the right hand/arm areas (i.e., sensations were felt either on the palm or the back of the hand, contralateral to the sonicated left hemisphere) (Table 2b). The individual-specific spatial distributions of sensations were illustrated in pseudo-color on the right hand (Fig. 2). It is interesting to note that the sensations felt from the fingers were either from a single digit/tip or from a group of two to five adjacent fingers (Additional file 1: Table S2). The sensation from the other locations (still all contralateral to the sonication), such as the wrist, forearm, elbow, and entire arm, were also reported. A few subjects (‘h1’-‘h3’) felt the sensations from the right leg (the knee or the calf) during the SI_FUS_ condition.

### Post-sonication safety profile of neurological and neuroradiological assessments

The neurological examination and MMSE, along with assessments of subject’s neuroradiological data, revealed no abnormal findings across all subjects. In the follow-up interviews conducted 8 weeks after the sonication, no discomforts or changes in the mental/physical status associated with the sonication procedure were reported.

## Discussion

In the present study, we demonstrated that image-guided, non-invasive transcranial FUS application to human SI and SII elicited various tactile sensations. We also showed the possibility of simultaneous acoustic stimulation of the SI and SII (proximal to each other), which has not been feasible with conventional non-invasive brain stimulation approaches such as TMS or tDCS. In terms of the type of sensations (Table 2; Additional file 1: Table S1), most of the elicited tactile sensations were similar to those from our previous study on acoustic stimulation of the SI [20]. The types of tactile sensations elicited from the SII sonication shared similarities with those elicited by electrical cortical stimulation of the SII [23]—cutaneous paresthesia (e.g., ‘tingling’, ‘light touch’, or ‘slight electric current’) or temperature sensations (e.g., ‘heat’ or ‘cold’). It may suggest that different brain stimulation modalities activating the same cortical areas (in this case, the SII) may result in the cognition of the similar tactile perception by engaging mutual cortical-level processing. Elicitations of the ‘warmth’ and ‘vibrotactile’ sensations were new findings, suggesting the possibility of creating a more diverse spectrum of tactile sensations.

Our initial hypothesis, in which selective FUS stimulation of the SII sub-regions (that are associated with different types of tactile sensations, i.e., vibrotactile, pressure, warmth, or coolness) would elicit corresponding tactile sensations, was rejected in the present study. We speculate that the FUS-mediated neurostimulation, achieved by FUS focus having the diameter of 6 mm and the length of 38 mm at FWHM (Fig. 1b), did not have sufficient spatial selectivity to stimulate the highly-overlapping sub-regions within the SII areas corresponding to differential tactile sensations [27, 37]. In addition, convoluted gyral structure in SII sub-regions [37, 38] may obscure the selective delivery of the FUS to these regions. The use of a FUS configuration, for example, a phased-array design of ultrasound system [9, 39] that has a smaller acoustic focus with wider aperture, would also be needed to provide greater spatial selectivity in acoustic stimulation. Another strategy to increase the spatial selectivity of FUS is to use higher acoustic frequencies [40], as the influence of the frequency on the size/shape of the focus is highlighted in the work by Pinton et al. [41]. The use of advanced brain mapping techniques, such as ultra-high field/spatial-resolution fMRI [42, 43], will also provide the ability to finely delineate sensation-specific sub-regions in the SII. Interestingly, subjects ‘h2’ and ‘h5’ reported matching types of sensations (such as ‘vibrotactile’, ‘pressure’, and ‘warmth’), which supports the feasibility of generating intended types of sensations when the sub-regions of the somatosensory areas are stimulated with greater spatial selectivity.

We found that the tactile sensations were reported from the hand/arm areas contralateral to the sonication across all FUS conditions (i.e., SI_FUS_, SII_FUS_, SI/SII_FUS_). In many occasions (*n* = 8), these sensations were localized in the palmar/dorsal side of the hand separately, or in a finger or in neighboring multiple fingers (Fig. 2; Additional file 1: Table S2). The topological distributions of these localized responses follow the major sensory innervation patterns of the radial, median, and ulnar nerves in the right upper extremity, which suggests spatially-selective stimulation of the relevant somatosensory areas (and nerve groups) by FUS. The sensations were also elicited away from the hand area (contralateral to the sonication), such as on the wrist, forearm, elbow, entire arm, and leg by a few subjects, which may be associated with the misaligned FUS stimulation (e.g., via acoustic refraction of the sonication at the skull) of the nearby somatosensory areas away from the hand SI or SII regions, whereby similar phenomena were seen from the previous study on the acoustic stimulation of the SI [20]. To reduce the experimental confounders induced by the acoustic attenuation/refraction at the skull, on-site numerical estimation of the acoustic propagation through/within the cranium can be utilized to estimate the in situ acoustic intensity as well as its spatial accuracy of the sonication prior to the FUS application.

In comparison of the response rates to that of our previous investigation on the acoustic stimulation of the SI [20], all three FUS conditions used in the present study showed similar levels of group-averaged response rates (Table 1). However, it is notable that about half of the subjects who reported elicited tactile sensations showed high levels of responsiveness (90–100%) in the SI_FUS_ condition (Table 1), while in the previous study [20], none achieved the high response rates of ≥90%. Although it is difficult to elucidate the exact causes for the improved stimulatory efficacy, we conjectured that the use of an increased level of incident acoustic energy (35 W/cm^2^ in the present study versus 3 W/cm^2^ in I_sppa_ previously) and the use of a longer SD (500 ms versus 300 ms), coupled with increased transcranial transmission rates due to the use of lower ultrasonic frequency (210 vs. 250 kHz), might have been contributing factors.

We observed several sessions that a few subjects did not report any elicited sensations (Table 1, indicated as ‘NR’). Considering varying FUS target locations and incident angles of the sonication beam for each session, with accompanying changes of skull thickness/shape on each sonication path, the attenuation and refraction of the FUS beam during the transcranial acoustic transmission may have reduced the level of in situ acoustic intensity at the intended target. Particularly for the non-responsive subject ‘h10’, the skull thickness on the sonication path to the SI was 7.8 mm (Additional file 1: Table S4), which was the greatest among the subjects. The skull may have attenuated/refracted a significant portion of acoustic energy to the level, perhaps below the threshold for excitation.

We noted that the response rates from the sonication greatly varied across the participants, ranging from 50 to 100% in one subject (‘h6’) to 10–35% in another subject (‘h1’) across the sonication sessions (Table 1). Similar degrees of individual variability in terms of responsiveness to the acoustic stimulation have been reported from our previous human study [20] as well as from large [21] and small animal models [17]. Although it is difficult to be ascertained for the causes to these phenomena, we hypothesized that the differential stimulatory sensitivity of the targeted neural substrates to the sonication may have contributed to the variability, which warrants further investigations. Interestingly, the presence of inter-subject variability in terms of responsiveness has been documented in studies of other brain stimulation modalities such as TMS [44].

We acknowledge that subjective measures on tactile sensations may be confounded by the individual’s attention to certain areas of the body [45, 46]. We attempted to address the attention-related sensations by blinding the participants on the nature of the stimulation (they were not expecting any sensations to begin with). Yet, the participants were able to identify the nature of the sensation (i.e., tactile) from the hand that was contralateral to the sonication. Due to the subtle and often unusual sensations (such as transient tingling and numbing sensations that disappear quickly upon each stimulatory events), unbiased characterization of the tactile sensations still poses as a challenging task [47]. More objective measures that are synchronized with the sonication timing, supported by the detection and characterization of the sensory evoked EEG potentials [20] in conjunction with randomized stimulation timing, may be used to strengthen the reliability of our findings. The use of well-designed sham/control condition will also be important for reducing the potential bias from the attention-related tactile illusion.

Use of the FUS technique allowed for simultaneous stimulation of ipsilateral SI and SII that are close to each other, which has not been achieved using other non-invasive brain stimulation methods. Although simultaneous stimulation of the SI and SII did not show any differential effects in terms of the tactile sensations or response rates, the ability to selectively stimulate these sensory areas may be applied to future investigations of chronic pain [23, 24], whereby the interactions of the SI and SII are important for perception and processing [48]. This possibility is also supported by previous studies of stimulation of the SII using TMS, which modulated the pain intensity among healthy volunteers [49, 50] or patients with chronic drug-resistant neuropathic pain [51]. In addition, FUS has been successfully delivered to the thalamic areas in humans [8, 10], whereby stimulation of the specific thalamic circuitries (e.g., including the ventral posterolateral nucleus of the thalamus) may also have potential to advance the pain-related studies. It is important to note, however, simultaneous sonication originating from two independent transducers may interfere with each other within the cranial cavity, and may subsequently form additional acoustic focus (or foci) having stimulatory potentials. In addition, acoustic reverberation [52] may also obscure the stimulation boundaries when multiple sonication beams are given proximal to each other. As these may confound stimulatory effects, caution is necessary when one aims to selectively simulate multiple brain regions. Also, accompanying acoustic simulations and corrective measures would help to reduce these confounders.

The neurostimulatory effects of FUS were transient and reversible, and the sonication procedure did not cause any adverse changes or discomforts in the mental/physical status across all subjects. Considering the average acoustic transmission rate of 20–25% at the intended targets [20] and a 50% duty cycle, it is estimated that 7.0–8.8 W/cm^2^ I_sppa_, corresponding to 3.5–4.4 W/cm^2^ I_spta_, was provided to the regional brain location. This estimated intensity range is slightly higher than the international electrotechnical commission (IEC) 60601 part 2 standard for therapeutic equipment limit of 3 W/cm^2^ I_spta_ [53]. Based on our past experience with sheep [21], as long as an excessive amount of stimulation is avoided, the intensity up to 13.4 W/cm^2^ I_sppa_ (in situ) does not cause any microscopic damage to the brain. However, this does not allow for the general application of the given parameters to human subjects and demands great caution when using higher acoustic intensity (and accompanying higher mechanical index (MI), while the current safety limit is set to 1.9 [53]). We estimated the potential thermal increase (ΔT) at the sonicated region of the brain by using the equation ΔT = 2αIt/ρ_b_C_p_ [54] = 2 × 0.005 cm^−1^ × 7.0 W/cm^2^ × 0.5 s/3.811 J/cm^3^ °C; where α = absorption coefficient [55], I = effective acoustic intensity (I_spta_) in the focal region considering the maximal transcranial acoustic transmission of 40% [20], t = sonication duration, ρ_b_ = density of the brain tissue [56], and C_p_ = specific heat of the brain tissue [56]. The estimated ΔT was 0.0092 °C, which was far below the thermal threshold that can derive either neurostimulatory effects or tissue damage [57, 58].

Along with promising safety data, the capability of FUS to selectively stimulate multiple brain regions, including those proximal to each other (such as ipsilateral SI and SII), would pave a new non-invasive way to study functional connectivity among neural substrates. Further studies employing fMRI for the assessment of network-level activations in the brain during FUS neuromodulation may help to reveal the causal relations between the region-specific brain functions of the stimulated neural substrates and the elicited cognitive/behavioral responses. The potential impact of FUS as a functional neuromodulation method awaits further evaluation across various disciplines from basic scientific studies to clinical applications.

## Conclusions

Simultaneous and regional acoustic stimulation of the SI/SII in the same hemisphere elicited various tactile sensations in the hand area contralateral to the sonication. The ability to selectively stimulate multiple human brain areas in spatially-restricted manner may offer unprecedented opportunity in the study of causal relationships between brain activity and subsequent efferent behaviors.

## Additional file

**Additional file 1.** **Table S1.** Sensations reported on the right hand/arm area. **Table S2.** Locations of the elicited sensations reported. **Table S3.** Subject’s descriptions of the elicited sensations (translated). **Table S4.** The skull thickness along each sonication path. **Figure S1.** An example of response time from subject ‘h6’ during the FUS experiment.

## Abbreviations

FUS: focused ultrasound
TMS: transcranial magnetic stimulation
tDCS: transcranial direct current stimulation
SI: the primary somatosensory areas
SII: the secondary somatosensory areas
fMRI: functional magnetic resonance imaging
DBS: deep brain stimulation
EpCS: epidural cortical stimulation
SEP: somatosensory evoked potentials
EEG: electroencephalography
IRB: institutional review board
CT: computed tomography
MMSE: the mini-mental state examination
FOV: field-of-view
TR: repetition time
TE: echo time
BOLD: blood oxygenation level dependent
OD: outer diameter
ROC: radius-of-curvature
PVA: polyvinyl alcohol
SD: sonication duration
ISI: inter-stimulation-interval
TBD: tone-burst-duration
PRF: pulse repetition frequency
FWHM: full-width at half-maximum
I_sppa_: spatial-peak pulse-average acoustic intensity
I_spta_: spatial peak temporal-average acoustic intensity
MI: mechanical index
