# Supplementary material for: Simultaneous acoustic stimulation of human primary and secondary somatosensory cortices using transcranial focused ultrasound
Source: BMC Neurosci. 2016 Oct 26;17:68. doi: 10.1186/s12868-016-0303-6 (PMC5081675; doi:10.1186/s12868-016-0303-6)
Supplement: Supplementary file 1 — Additional file 1. Table S1. Sensations reported on the right hand/arm area. Table S2. Locations of the elicited sensations reported. Table S3. Subject’s descriptions of the elicited sensations (translated). Table S4. The skull thickness along each sonication path. Figure S1. An example of response time from subject ‘h6’ during the FUS experiment. [file 12868_2016_303_MOESM1_ESM.docx]

**Supplementary Information**

Simultaneous acoustic stimulation of human primary and secondary somatosensory cortices using transcranial focused ultrasound

Wonhye Lee^1,2^, Yong An Chung^1^, Yujin Jung^1^, In-Uk Song^1^, and Seung-Schik Yoo^1,2*^

^1^Incheon St. Mary’s Hospital, The Catholic University of Korea, Incheon, Korea

^2^Department of Radiology, Brigham and Women’s Hospital, Harvard Medical School, Boston, MA, USA

Supplementary Tables

**Table S1. Sensations reported on the right hand/arm area**. NR denotes non-responsive cases. V, vibrotactile; P, pressure; W, warmth; C, coolness.

|  | **SI_FUS_** | **SII_FUS_** | | | | **SI/SII_FUS_** | | | |
| --- | --- | --- | --- | --- | --- | --- | --- | --- | --- |
| **ID** |  | **V** | **P** | **W** | **C** | **V** | **P** | **W** | **C** |
| **h1** | Tingling, Feeling of weak electrical current flow | Tingling, Feeling of weak electrical current flow | Tingling, Feeling of weak electrical current flow | Feeling of weak electrical current flow | Numbness, Feeling of weak electrical current flow | Tingling, Feeling of electrical current flow | Numbness, Feeling of weak electrical current flow | Feeling of weak electrical current flow | Numbness, Feeling of weak electrical current flow |
| **h2** | Tingling, Warmth | Warmth | Warmth | Warmth | Tingling | Tingling, Warmth | Tingling | Warmth | Warmth |
| **h3** | Tingling, Numbness | Tingling, Feeling of weak electrical current flow | Tingling | Tingling | Numbness | Tingling | Tingling, Feeling of weak electrical current flow | Tingling | Tingling, Feeling of weak electrical current flow |
| **h4** | Tingling, Numbness, Feeling of weak  electrical current flow | NR | Tingling | NR | NR | Tingling | Tingling | NR | NR |
| **h5** | Heaviness/ Pressure, Warmth | Warmth, Coolness, Pressure (Touching) | NR | Warmth | Tingling, Pressure | Vibrotactile | Pressure, Heaviness | Feeling of weak electrical current flow | Numbness |
| **h6** | Tingling,  Feeling of weak electrical current flow | Tingling, Feeling of weak electrical current flow | Tingling, Feeling of weak electrical current flow | Tingling | Tingling, Feeling of weak electrical current flow | Tingling, Feeling of weak electrical current flow | Tingling | Tingling | Tingling |
| **h7** | Numbness,  Brushing | Feeling of weak electrical current flow | NR | Numbness | Feeling of weak electrical current flow | Feeling of weak electrical current flow | Numbness | Feeling of weak electrical current flow | Numbness |
| **h8** | Tingling | Numbness | Numbness | NR | NR | NR | NR | NR | NR |
| **h9** | Tingling, Heaviness/ Pressure, Feeling of weak  electrical current flow, Vibrotactile | NR | NR | Tingling, Feeling of weak electrical current flow | Pressure | Feeling of weak electrical current flow | Numbness | Tingling | Vibrotactile, Numbness, Tingling, Feeling of weak electrical current flow |
| **h10** | NR | NR | NR | NR | NR | NR | NR | NR | NR |

**Table S2.** **Locations of the elicited sensations reported.** NR denotes non-responsive cases. V, vibrotactile; P, pressure; W, warmth; C, coolness

|  | **SI_FUS_** | **SII_FUS_** | | | | **SI/SII_FUS_** | | | |
| --- | --- | --- | --- | --- | --- | --- | --- | --- | --- |
| **ID** |  | **V** | **P** | **W** | **C** | **V** | **P** | **W** | **C** |
| **h1** | Hand, Finger (1), Leg (Calf) | Fingers (all, tips), Arm | Forearm | Hand (back), Wrist (dorsal) | Forearm (radial side) | Hand | Forearm | Hand (palm), Fingers (all) | Hand |
| **h2** | Hand (palm), Finger (1),  Leg | Hand | Hand (palm) | Hand (palm) | Fingers (all) | Hand (palm) | Fingers (2,3,4 tips) | Hand | Hand |
| **h3** | Hand (palm), Fingers (2,3, tips),  Leg (Knee) | Hand, Fingers (1,2,3,4,5 tips), Forearm, Elbow | Hand (palm), Fingers (all) | Forearm, Elbow | Hand (palm), Fingers (all) | Finger (3, tip and joints), Wrist (dorsal) | Hand (palm), Fingers (all) | Elbow, Forearm | Hand (palm), Finger (1) |
| **h4** | Forearm, Elbow | NR | Elbow | NR | NR | Elbow | Elbow | NR | NR |
| **h5** | Fingers (all, tips) | Wrist (dorsal) | NR | Forearm (radial side) | Finger (5) | Wrist (dorsal) | Forearm, Elbow | Hand (palm) | Finger (5) |
| **h6** | Hand (entire, back), Fingers (2,3) | Hand (back) | Hand (palm), Fingers (all) | Hand (palm) | Hand (back) | Hand (back) | Hand | Hand | Hand (back) |
| **h7** | Wrist (dorsal), Forearm, Elbow | Hand | NR | Wrist (dorsal) | Hand (back), Fingers (2,3) | Hand (back), Fingers (4, 5), Elbow | Hand (palm), Fingers (3,4,5) | Hand (palm), Finger (5) | Hand (ulnar side), Finger (5) |
| **h8** | Hand, Forearm | Hand (palm), Fingers (all), Arm | Hand (palm), Fingers (all tips) | NR | NR | NR | NR | NR | NR |
| **h9** | Hand,  Finger (3),  Arm (entire, inside) | NR | NR | Hand (palm), Fingers (4, 5) | Arm | Hand (palm), Fingers (All), Arm | Elbow, Arm (upper), | Elbow,  Arm (upper), | Hand |
| **h10** | NR | NR | NR | NR | NR | NR | NR | NR | NR |

**Table S3. Subject’s descriptions of the elicited sensations (translated).** NR denotes non-responsive cases. V, vibrotactile; P, pressure; W, warmth; C, coolness.

| **ID** | **Session** | **Subject’s descriptions of the elicited sensations (translated)** |
| --- | --- | --- |
| **h1** | **SI_FUS_** | I felt weak tingling sensation and the feeling of weak electrical flow from my right hand, including the thumb. I also felt similar sensation from the calf of my right leg. |
|  | **SII_FUS_-V** | I felt tingling, combined with electrical flow-like sensations, from my right arm and hand, including all its fingers. |
|  | **SII_FUS_-P** | I felt tingling, combined with electrical flow-like sensation from my right forearm. The sensation was strong and distinct. |
|  | **SII_FUS_-W** | I felt electrical flow from the back of my right hand as well as the right wrist (with gestures pointing to the dorsal side). The sensations were strong. |
|  | **SII_FUS_-C** | I felt bit of numbing sensation mixed with weak electrical flow from the radial side of the forearm (with hand-gestures indicating the location ) |
|  | **SI/SII_FUS_-V** | I felt very weak electrical flow and tingling sensation from my right hand. |
|  | **SI/SII_FUS_-P** | I felt very weak electrical flow from my right hand. I also felt numbing-like sensation, too. |
|  | **SI/SII_FUS_-W** | I felt weak electrical flow from my right palm, radiating to all of my fingers. |
|  | **SI/SII_FUS_-C** | I felt weak electrical flow from my right hand. I also felt numbing sensations. |
| **h2** | **SI_FUS_** | My right palm and thumb got tingling and warmer, but it did not hurt. I also felt the same sensation from my right leg. |
|  | **SII_FUS_-V** | I felt warmth from my right hand. The sensation got stronger, but I do not feel it now. |
|  | **SII_FUS_-P** | I felt warmth in my right palm |
|  | **SII_FUS_-W** | I felt warm and hot sensations in my right palm |
|  | **SII_FUS_-C** | I am not certain, but I felt tingling sensations from all my right fingers. |
|  | **SI/SII_FUS_-V** | I felt tingling and warmth from my right palm. |
|  | **SI/SII_FUS_-P** | I felt weak tingling sensation from my palm side of these finger tips (with gestures indicating the middle/index/ring fingers) |
|  | **SI/SII_FUS_-W** | I certainly felt warmth from my right hand. |
|  | **SI/SII_FUS_-C** | I felt warmth from my right hand. |
| **h3** | **SI_FUS_** | I felt strong tingling and numbness from the tip of my right index and middle fingers. I also felt these sensations from my right palm and right leg, but only weaker. |
|  | **SII_FUS_-V** | My right elbow, forearm, and hand were tingling. It was like having a weak electrical stimulation. |
|  | **SII_FUS_-P** | My right palm and all right fingers were tingling periodically. |
|  | **SII_FUS_-W** | My right elbow and forearm were tingling periodically. |
|  | **SII_FUS_-C** | I felt the numbing sensation from my right palm, including all fingers of the right hand. |
|  | **SI/SII_FUS_-V** | My right middle finger tip, joins, and back of my wrist were tingling periodically. |
|  | **SI/SII_FUS_-P** | I felt spreading feeling of tingling sensation, from right palm to all its fingers. It did not hurt, but it was uncomfortable. |
|  | **SI/SII_FUS_-W** | I felt tingling sensation from my right elbow and forearm. |
|  | **SI/SII_FUS_-C** | I felt tingling sensation and weak electrical flow-like sensations, spreading from the root to tip of my right thumb. |
| **h4** | **SI_FUS_** | The feeling I had was mixture of tingling, numbness, and very weak version of the electric current shock. It is similar to the sensation when our elbow is hit on a funny bone. The sensations were coming from the right elbow and forearm. |
|  | **SII_FUS_-V** | NR |
|  | **SII_FUS_-P** | I felt tingling sensation from my right elbow. |
|  | **SII_FUS_-W** | NR |
|  | **SII_FUS_-C** | NR |
|  | **SI/SII_FUS_-V** | I felt tingling sensation from my right elbow. It was pretty strong. |
|  | **SI/SII_FUS_-P** | I felt tingling sensation from my right elbow. |
|  | **SI/SII_FUS_-W** | NR |
|  | **SI/SII_FUS_-C** | NR |
| **h5** | **SI_FUS_** | I felt someone squeezing my right hand and fingers. I also felt warmth that spread from my right hand. |
|  | **SII_FUS_-V** | I felt the warmth from my back of my wrist, but later I felt cool sensation from the same location. I also felt someone was touching the same area. |
|  | **SII_FUS_-P** | NR |
|  | **SII_FUS_-W** | I felt warmth from my right forearm (with gestures indicating its radial side). |
|  | **SII_FUS_-C** | I felt someone is poking my right little finger. It did not hurt, but I felt pressure on it. |
|  | **SI/SII_FUS_-V** | I felt weak vibration from the back of my right wrist. |
|  | **SI/SII_FUS_-P** | I felt someone pressing my right elbow and forearm, |
|  | **SI/SII_FUS_-W** | I felt weird sensation that something flows from inside of my right palm to my fingers. |
|  | **SI/SII_FUS_-C** | My right pinky finger got numb periodically. |
| **h6** | **SI_FUS_** | I felt tingling and weak electrical current flow spreading from right hand (with gestures on back of the hand and index/middle fingers). I could certainly feel them. |
|  | **SII_FUS_-V** | I felt tingling and spreading weak electrical current flow fin the back of my right hand. |
|  | **SII_FUS_-P** | I felt tingling and spreading weak electrical current flow from all the right fingers, especially stronger from the thumb. |
|  | **SII_FUS_-W** | I felt tingling from my right palm. |
|  | **SII_FUS_-C** | I felt tingling and spreading weak electrical current flow from back of my right hand. |
|  | **SI/SII_FUS_-V** | I felt tingling and spreading very weak electrical current flow from back of my right hand. |
|  | **SI/SII_FUS_-P** | I felt tingling from my right hand. |
|  | **SI/SII_FUS_-W** | I felt tingling from my right hand. |
|  | **SI/SII_FUS_-C** | I felt tingling from my back of my right hand. |
| **h7** | **SI_FUS_** | I felt something brushing against back of the right wrist and elbow, including the right forearm. I also felt little numbness from the areas as well. |
|  | **SII_FUS_-V** | I felt electrical flow-like sensation from my right hand. |
|  | **SII_FUS_-P** | NR |
|  | **SII_FUS_-W** | I feel weak numbness from back of my right wrist (with hand gesture indicating the dorsal side) |
|  | **SII_FUS_-C** | I felt weak electrical current-like sensation that flows from right index/middle fingers to the right hand (on the dorsal side). |
|  | **SI/SII_FUS_-V** | I felt weak electrical current flow that radiated from the back of right hand/elbow to the ring/little fingers. |
|  | **SI/SII_FUS_-P** | I felt numbness, although weak, from the right palm, and middle/ring/little fingers. |
|  | **SI/SII_FUS_-W** | I felt weak electrical current flow originating from the right little finger, radiating to the back of the right hand. |
|  | **SI/SII_FUS_-C** | I felt numbness from the right little finger and the ulnar side of the right hand (indicating with a hand gesture) |
| **h8** | **SI_FUS_** | I felt weak tingling sensation from the right hand, including the forearm. |
|  | **SII_FUS_-V** | I felt numbing sensation from the both sides of upper arms, right palm and all right fingers. |
|  | **SII_FUS_-P** | I felt numbness from the all right finger tips and palm. |
|  | **SII_FUS_-W** | NR |
|  | **SII_FUS_-C** | NR |
|  | **SI/SII_FUS_-V** | NR |
|  | **SI/SII_FUS_-P** | NR |
|  | **SI/SII_FUS_-W** | NR |
|  | **SI/SII_FUS_-C** | NR |
| **h9** | **SI_FUS_** | I felt tingling sensation mixed with heaviness and bit of pressure on the radial (with hand gesture) side of my right arm, including my right hand and finger tips. It was also like an electrical current flow that passes through the arm. I also felt a bit of vibratory sensation as well. |
|  | **SII_FUS_-V** | NR |
|  | **SII_FUS_-P** | NR |
|  | **SII_FUS_-W** | I felt tingling and electrical current flow (weak) from the right palm and ring/little fingers. |
|  | **SII_FUS_-C** | I felt something pressing against my right arm in a periodic fashion. |
|  | **SI/SII_FUS_-V** | I felt sensation of weak electric current flow from ventral side (indicating with hand gesture) of the right upper arm, hand and fingers. |
|  | **SI/SII_FUS_-P** | I felt weak numbness from the right elbow and upper arm |
|  | **SI/SII_FUS_-W** | I felt weak tingling from the right upper arm and elbow. |
|  | **SI/SII_FUS_-C** | I felt weak vibration, numbness, and tingling from the right hand. I felt something similar to electrical current flow from the same area. |
| **h10** | **SI_FUS_** | NR |
|  | **SII_FUS_-V** | NR |
|  | **SII_FUS_-P** | NR |
|  | **SII_FUS_-W** | NR |
|  | **SII_FUS_-C** | NR |
|  | **SI/SII_FUS_-V** | NR |
|  | **SI/SII_FUS_-P** | NR |
|  | **SI/SII_FUS_-W** | NR |
|  | **SI/SII_FUS_-C** | NR |

**Table S4**. **The skull thickness along each sonication path**. V, vibrotactile; P, pressure; W, warmth; C, coolness.

| **ID** | **Skull thk to SI (mm)** | **Skull thk to SII (mm)** | | | |
| --- | --- | --- | --- | --- | --- |
|  |  | **V** | **P** | **W** | **C** |
| **h1** | 4.7 | 2.8 | 2.8 | 2.8 | 2.8 |
| **h2** | 4.0 | 1.9 | 1.9 | 2.3 | 2.3 |
| **h3** | 5.3 | 3.8 | 3.9 | 4.0 | 4.0 |
| **h4** | 3.4 | 3.9 | 3.9 | 4.0 | 3.9 |
| **h5** | 4.2 | 4.7 | 4.7 | 3.8 | 3.8 |
| **h6** | 6.8 | 3.1 | 3.1 | 3.1 | 3.1 |
| **h7** | 5.5 | 2.8 | 3.8 | 3.9 | 2.8 |
| **h8** | 5.6 | 3.9 | 3.9 | 4.7 | 3.9 |
| **h9** | 3.4 | 2.8 | 2.8 | 3.9 | 2.8 |
| **h10** | 7.8 | 3.8 | 4.0 | 3.8 | 4.7 |
| Mean | 5.1 | 3.4 | 3.5 | 3.6 | 3.4 |
| s.d. | 1.4 | 0.8 | 0.8 | 0.7 | 0.8 |

Supplementary Figure


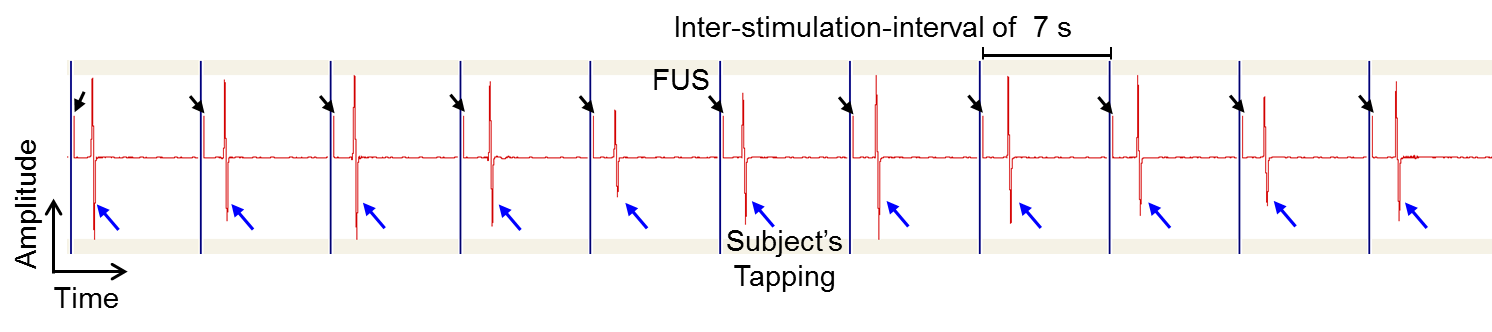


**Figure S1. An example of response time from subject ‘h6’ during the FUS experiment.** Black arrows indicate the timing of FUS delivered to the SI and SII either separately or simultaneously, and blue arrows indicate the tapping from the participants when the tactile sensory sensations were elicited by the neurostimulatory sonication. The time gap (*i.e.*, inter-stimulation-interval) between sonication events was 7 s, and a total of 20 FUS events were given during each sonication condition of SI_FUS_, SII_FUS_, and SI/SII_FUS_, respectively. These data were used for the derivation of the response rate of the tactile sensations elicited across the subjects.
